# Mathematical Model for Skin Pain Sensation under Local Distributed Mechanical Compression for Electronic Skin Applications

**DOI:** 10.3390/mi13091402

**Published:** 2022-08-26

**Authors:** Dongcan Ji, Yingli Shi, Jiayun Chen, Zhao Zhao, Guozhong Zhao

**Affiliations:** 1 Institute of Solid Mechanics, Beihang University (BUAA), Beijing 100191, China; 2 School of Materials and Energy, University of Electronic Science and Technology of China (USETC), Chengdu 610054, China; 3 State Key Laboratory of Structural Analysis for Industrial Equipment, Department of Engineering Mechanics, Dalian University of Technology, Dalian 116024, China

**Keywords:** skin pain sensation, electronic skin, mechanical compression, revised Hodgkin–Huxley model, gate control theory

## Abstract

Skin pain resulting from mechanical compression is one of the most common pains in daily life and the indispensable information for electronic skin to perceive external signals. The external mechanical stimuli are transduced into impulses and transmitted via nerve fiber, and finally, the sensation is perceived via the procession of the nerve system. However, the mathematical mechanism for pain sensation due to mechanical stimuli remains unclear. In this paper, a mathematical model for skin pain sensation under compression is established, in which the Flament solution, the revised Hodgkin–Huxley model, and the mathematical model gate control theory are considered simultaneously. The proposed model includes three parts: a mechanical model of skin compression, a model of transduction, and a model of modulation and perception. It is demonstrated that the pain sensation degree increases with the compression amplitude and decreases with deeper nociceptor location in the skin. With the help of the proposed model, the quantitative relationship between compression pain sensation and external mechanical stimuli is revealed, which has a significant benefit in promoting the design and mechanism research of electronic skin with pain perception function.

## 1. Introduction

**Figure 1:**
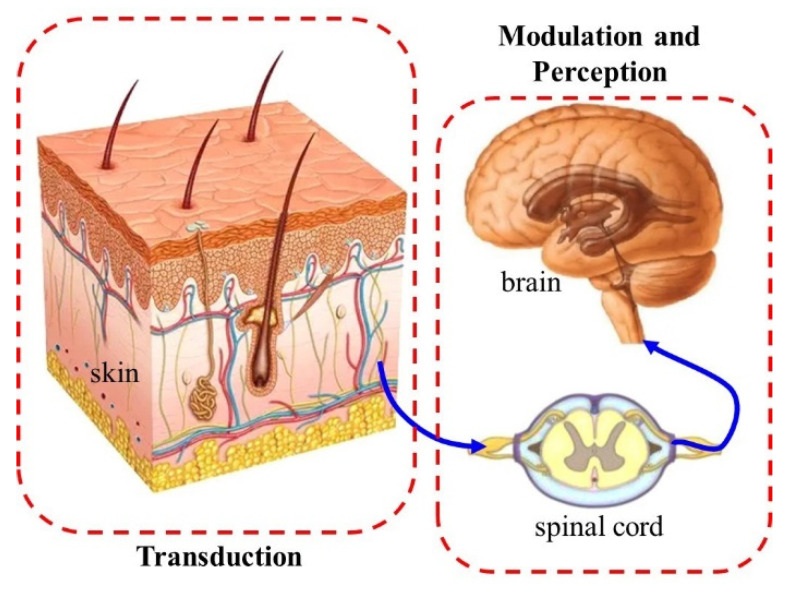
Diagram of the skin pain model.

Skin, the largest human organ, wraps the surface of the body and is in direct touch with the external environment [1]. It has many essential functions including protecting, excreting, and thermoregulation [2], and one of the most important functions is sensing external stimuli, including mechanical [3,4] and thermal stimuli [5,6,7]. As illustrated in Figure 1, external mechanical stimuli could be perceived as the sensation of touching with the transduction of skin and the modulation of the nerve system [8]. However, extreme mechanical stimuli will cause unpleasant pain sensations, or even damage the skin [9,10].

There were two main types of theories explaining the pain sensation mechanism before the 1960s. The first one is the specific theory, which indicates that each kind of pain owns a specific pathway to the brain [11,12]. The second one is the pattern theory, which proposes that the pain information transmitted into the brain is coded via the spatiotemporal pattern of the impulses [13,14]. However, both these theories have their shortcomings: the specific pain theory cannot explain the fact that pain can be relieved by rubbing the injured skin, and the decoding mechanism for the pattern theory remains ambiguous. The proposition of gate control theory (GCT) [15] makes it possible to explain many experimental phenomena, but GCT cannot elucidate the relationship between external stimuli and the nerve impulse.

Although pain has been studied from the molecular level to the level of the entire nervous system for a long period, the computation models of pain sensation are still limited [16,17]. Researchers have developed mathematical models at different levels: molecular [18,19], cellular [20], and neuron network levels [11,21,22]. Britton et al. [20] established a mathematical model for GCT and used it to explain the quality of pain. Xu et al. [5,8,16] have systematically studied the thermal pain sensation. By combining the transduction, transmission, modulation and perception process, the temperature, thermal stress, and chemical burn are all taken into consideration in thermal pain analysis. Based on Xu’s theory, Yin et al. [9] studied the skin pain sensation under the heating of epidermal electronic devices.

There are three main pain stimuli: thermal, mechanical, and chemical stimulation [16]. Among these, mechanical stimulation has drawn little attention in pain analysis; there are few pieces of research about the evaluation of the human sensation under the tactile sensing of electrical skin. The aforementioned issues motivated us to study the superficial acute pain sensation due to mechanical stimuli. In this paper, firstly, the mechanical model of compression on the skin is investigated and the theoretical stress distribution is obtained based on the Flament solution. Then, setting the stress as an input, the pain sensation is evaluated based on the modified Hodgkin–Huxley model and the gate control theory. The theoretical stress distribution is verified by finite element analysis (FEA). The influences of compression magnitude and nociceptor location are also investigated.

## 2. Modeling and Analysis

There are three main types of pain: nociceptive pain, inflammatory pain, and neuropathic pain [16]. Nociceptive pain has been studied from different aspects before. Figure 1 is a schematic of the pain sensation process, including (1) transduction: the different kinds of outside stimuli are converted into nerve impulses by receptors (nociceptors) in the skin; (2) transmission: the nerve impulses, which carry the information of stimuli, transmit to the dorsal horn through nerve fibers; (3) modulation: downward inhibition and facilitation of nociceptive transmission; (4) perception: the evaluation of signals received in higher-order structures of the nervous system. Thus, a mathematical model of compression pain is established in this paper, which consists of three parts: the mechanical model of skin compression, the model of transduction, and the model of modulation and perception. The time delay caused by the transmission is omitted in this paper. In the following parts of this section, the details of the model will be demonstrated.

### 2.1. Mechanical Model of Skin Compression

**Figure 2:**
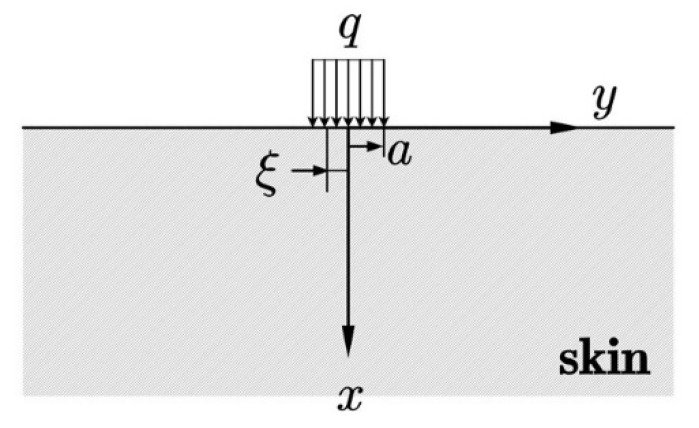
The mechanical model of compression on the skin.

Similar to thermal pain, which has been studied by Yin [9] before, compression pain is determined by stress at the location of the nociceptors, instead of the surface of the skin. As compression is applied on the surface of the skin (Figure 2), the sensation of touching will first be generated. With the growth of the compression amplitude, the stress on the tissue will increase and reach a critical value [9], which may lead to the sensation of pain. Mechanical pain is not only an unpleasant feeling but also potentially damaging to the skin and nervous system [10]. Thus, the mechanical model of skin is developed in this part, where the skin is regarded as a two-dimensional semi-infinite solid for simplicity, as demonstrated in Figure 2. A uniform compression with amplitude *q* and length 2*a* is applied on the top surface of the skin. To obtain the stress field of skin, the Flament answer to the semi-infinite plane strain problem is utilized here [23]:

(1) $$\sigma_{x}=-\frac{2}{\pi }\int_{-a}^{a}\frac{q\left(\xi \right)x^{3}d\xi }{{\left[x^{2}+{\left(y-\xi \right)}^{2}\right]}^{2}}$$

(2) $$\sigma_{y}=-\frac{2}{\pi }\int_{-a}^{a}\frac{q\left(\xi \right)x{\left(y-\xi \right)}^{2}d\xi }{{\left[x^{2}+{\left(y-\xi \right)}^{2}\right]}^{2}}$$

(3) $$\tau_{xy}=-\frac{2}{\pi }\int_{-a}^{a}\frac{q\left(\xi \right)x^{2}\left(y-\xi \right)d\xi }{{\left[x^{2}+{\left(y-\xi \right)}^{2}\right]}^{2}}$$

The strain field of skin can be acquired by integrating *ξ* in Equation (1) from −*a* to *a*. When *y* belongs to different intervals: (*−a*, *a*) or (*a*, ∞)∪(−∞, −*a*), the integration results are distinct, noting that it is compression pain that is being investigated, and *y* stands for the position of the nociceptor on the skin [19]. Hence, it is reasonable to assume that the nociceptor locates at the *x*-axis in compression pain, for the convenience of the analysis, leading the integration in Equations (1)–(3) to:

(4) $$\sigma_{x}=-\frac{q}{\pi {\left(a^{2}+x^{2}\right)}^{2}}\left[2\arctan\left(\frac{a}{x}\right)a^{4}+4\arctan\left(\frac{a}{x}\right)a^{2}x^{2}+2\arctan\left(\frac{a}{x}\right)x^{4}+2a^{3}x+2ax^{3}\right]$$

(5) $$\sigma_{y}=-\frac{q}{\pi {\left(a^{2}+x^{2}\right)}^{2}}\left[2\arctan\left(\frac{a}{x}\right)a^{4}+4\arctan\left(\frac{a}{x}\right)a^{2}x^{2}+2\arctan\left(\frac{a}{x}\right)x^{4}-2a^{3}x-2ax^{3}\right]$$

(6) $$\tau_{xy}=0$$

Since there is no shear stress, *σ_x_* and *σ_y_* are the two principal stresses at the location of the nociceptors with

(7) $$\sigma_{x}<\sigma_{y}<0$$

where *σ_x_* is the minor principal stress and *σ_y_* is the major principal stress on the *x*-axis. 

### 2.2. Model of Transduction

#### Current and Frequency Modulation

**Figure 3:**
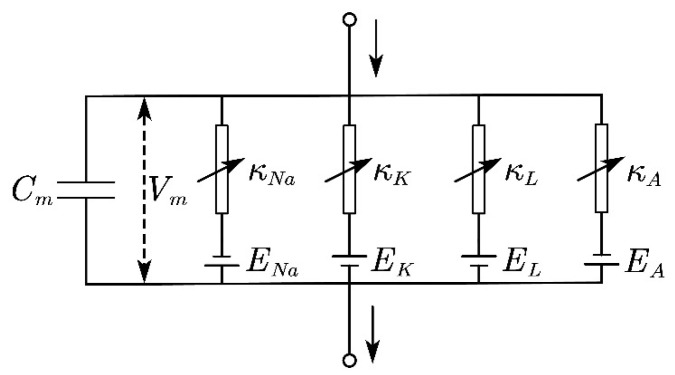
Modified Hodgkin–Huxley (H–H) model.

When the skin is stimulated by the compression, the nociceptors trigger potential action as a result of ion transportation across the cell membrane of the neuron [19]. The membrane potential of the nerve excitation can be described by the revised Hodgkin–Huxley (H–H) model [18,24,25], as illustrated in Figure 3. The total current consists of the transportation of ions across the corresponding ion channel and the current that charges the membrane capacity, which gives:

(8) $$C_{m}\frac{dV_{m}}{dt}=I_{\mathrm{mech}}+I_{\mathrm{shift}}-\left(I_{Na}+I_{K}+I_{A}+I_{L}\right)$$

where *V_m_* (mV) stands for the membrane potential of the nociceptors, and *t* (ms) represents the time. *C_m_* = 1 μF/cm^2^ is the membrane capacitance per unit area [9]. *I_Na_*, *I_K_*, *I_A_*, and *I_L_* correspond to the current induced by the sodium ions (Na^+^), potassium ions (K^+^), fast transient K^+^, and leakage current component. *I*_mech_ is the current induced by the mechanical stimuli, which is revealed to be a function of the stress at the location of the nociceptor:

(9) $$I_{\mathrm{mech}}=\left[C_{m1}\exp\left(\frac{\left(\sigma -\sigma_{t}\right)/\sigma_{t}}{C_{m2}}\right)+C_{m3}\right]\times H\left(\sigma -\sigma_{t}\right)$$

Here, *σ*_t_ = 20 kPa [26] is the mechanical threshold and *H*(*x*) is the Heaviside function. *C*_m1_ = 2 μA/cm^2^, *C*_m2_ = 2, *C*_m3_ = −1 μA/cm^2^ are the mechanical stimuli-related constants and *I*_shift_ = 8.1 mA is the current to guarantee the action potential when *σ > σ*_t_ [27].

The ionic current *I_Na_*, *I_K_*, *I_A_*, and *I_L_* driven by the membrane potential could be described as:

(10) $$I_{Na}=\kappa_{Na}m^{3}h\left(V_{m}-E_{Na}\right)$$

(11) $$I_{K}=\kappa_{K}n^{4}\left(V_{m}-E_{K}\right)$$

(12) $$I_{A}=\kappa_{A}A^{3}B\left(V_{m}-E_{A}\right)$$

(13) $$I_{L}=\kappa_{L}\left(V_{m}-E_{L}\right)$$

where *E_Na_* = 55 mV, *E_K_* = −72 mV, *E_A_* = −75 mV, and *E_L_* = −17.5 mV are the reversal potentials as demonstrated in Figure 3. *κ_i_* represents different ionic conductance here and *κ_Na_* = 120 mS/cm^2^, *κ_K_* = 20 mS/cm^2^, *κ_A_* = 47.7 mS/cm^2^, and *κ_L_* = mS/cm^2^ [9]. The *m*, *n*, *h*, *A*, and *B* are the gating variables satisfy:

(14) $$\tau_{x}\frac{dx}{dt}+x=x_{\infty }$$

where *x* represents the gating variables. For *x* = *m*, *h*, *n*, the expressions of *τ_x_* and *x_∞_* are given as follows:

(15) $$x_{\infty }=\frac{\alpha_{x}}{\alpha_{x}+\beta_{x}},\;\tau_{x}=\frac{1}{\alpha_{x}+\beta_{x}}$$

(16) $$\alpha_{m}=\frac{\left(V_{m}+29.7\right)/10}{1-\exp\left(-\left(V_{m}+29.7\right)/10\right)},\;\beta_{m}=4\exp\left(-\left(V_{m}+54.7\right)/18\right)$$

(17) $$\alpha_{h}=0.07\exp\left(-\left(V_{m}+48\right)/20\right),\;\beta_{h}=\frac{1}{1+\exp\left(-\left(V_{m}+18\right)/10\right)}$$

(18) $$\alpha_{n}=\frac{\left(V_{m}+45.7\right)/100}{1-\exp\left(-\left(V_{m}+45.7\right)/10\right)},\;\beta_{n}=0.125\exp\left(-\left(V_{m}+55.7\right)/80\right)$$

For *x* = *A*, *B*, the expressions for *τ_x_* and *x_∞_* are:

(19) $$\begin{array}{ll} \tau_{A} & =0.3632+\frac{1.158}{1+\exp\left(\left(V_{m}+55.96\right)/20.12\right)},\\ A_{\infty } & ={\left(0.0761\times \frac{\exp\left(\left(V_{m}+94.22\right)/31.84\right)}{1+\exp\left(\left(V_{m}+1.17\right)/28.93\right)}\right)}^{1/3};\; \end{array}$$

(20) $$\begin{array}{ll} \tau_{B} & =1.24+\frac{2.678}{1+\exp\left(\left(V_{m}+50\right)/16.027\right)},\\ B_{\infty } & ={\left(\frac{1}{1+\exp\left(\left(V_{m}+53.3\right)/14.54\right)}\right)}^{4}. \end{array}$$

*V_m_* can be solved by substituting Equations (9)–(20) into Equation (8). Instead of the amplitude, the frequency of *V_m_* represents the intensity of mechanical stimuli, which is utilized in the analysis of modulation and perception in the following section. 

### 2.3. Model of Modulation and Perception

**Figure 4:**
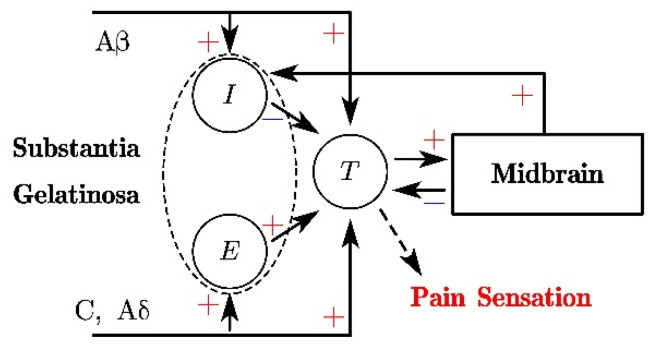
The schematic of the mathematical model of gate control theory (GCT).

There are various theories explaining the relationship between neural excitation and pain sensation. Among them, the gate control theory, which is proposed by Melzack [15], is one of the most successful theories, which precisely describes the modulation and perception procedure of skin pain sensation. Britton et al. [20] give the mathematical model of GCT, which is schematically illustrated in Figure 4. The mathematical descriptions are given as:

(21) $$0.7\frac{dV_{i}}{dt}=-\left(V_{i}+70\right)+60\tanh\left(\theta_{li}x_{l}\right)+40\tanh\left(f_{b}\left(V_{b}\right)\right)$$

(22) $$0.7\frac{dV_{e}}{dt}=-\left(V_{e}+70\right)+40\tanh\left(\theta_{se}x_{s}\right)\left[1+3\tanh\left(4f_{e}\left(V_{e}\right)\right)\right]$$

(23) $$\begin{array}{ll} 0.7\frac{dV_{t}}{dt} & =-\left(V_{t}+70\right)+40\tanh\left(\left(1-\theta_{se}\right)x_{s}\right)+40\tanh\left(\left(1-\theta_{li}\right)x_{l}\right)\\ & +40\tanh\left(f_{e}\left(V_{e}\right)\right)-40\tanh\left(f_{i}\left(V_{i}\right)\right)-40\tanh\left(f_{b}\left(V_{b}\right)\right) \end{array}$$

(24) $$0.7\frac{dV_{b}}{dt}=-\left(V_{b}+70\right)+40\tanh\left(f_{t}\left(V_{t}\right)\right)$$

where the *V_i_* and *V_e_* are the potentials of the inhibitory substantia gelatinosa (SG) and excitatory SG cells, and *V_t_* and *V_b_* stand for the potential of central transmission cell (T-cell) and midbrain. The subscript *l* and *s* represent the large fibers (C, Aδ) and small fibers (Aβ), respectively. *θ_li_* and *θ_se_* represent the proportion of excitation transmitted to inhibitory and excitatory SG cells through large and small nerve fibers. Both *θ_li_* and *θ_se_* are taken as 0.8 in this paper. *x_i_* denotes the frequency of the membrane potential transmitted on the fiber, and the definition of function *f_j_* (*V_j_*) is

(25) $$f_{j}\left(V_{j}\right)=\left(\frac{V_{j}-V_{thr}}{-V_{j0}}\right)\times H\left(V_{j}-V_{thr}\right)$$

Here, the subscript *j* represents *i*, *e*, *t*, *b* in Equations (21)–(24). *V_j_*_0_ = −70 mV is the initial membrane potential, and *V_thr_* = −55 mV is the threshold potential value for the pain sensation [9]. The output of T-cell *V_t_* is in direct relation to the pain sensation because the noxious signal is transmitted to the cortex when *V_t_* exceeds the threshold value. Note, it has been demonstrated that intense noxious stimuli information is transmitted through small fibers while the slight stimuli information is carried by large fibers. Since compression pain is analyzed in this paper, the *x_l_* and *x_s_* are taken to be zero and the frequency of *V_m_*, respectively [9].

## 3. Results and Discussion

### 3.1. Stress Distributions in the Skin

The strain distributions of the skin in Equations (4)–(6) were verified by finite element analysis. A two-dimensional rectangular solid with 10 m × 5 m was established in ABAQUS software, which was large enough to simulate semi-infinity. A pressure *q* = 20 kPa with radius *a* = 10 mm was applied on the top surface of the skin and the other surfaces of the skin were fixed. Young’s modulus and Poisson’s ratio were set to be *E* = 20 kPa and *ν* = 0.3 [6,28]. The six-node triangular CPS6M element was chosen to discretize the model. The size of the elements was controlled in the range from 0.005 m to 0.2 m with a fine mesh around the compressed region. The number of the elements was 3524, which was large enough to guarantee the convergence of the FEA model.

**Figure 5:**
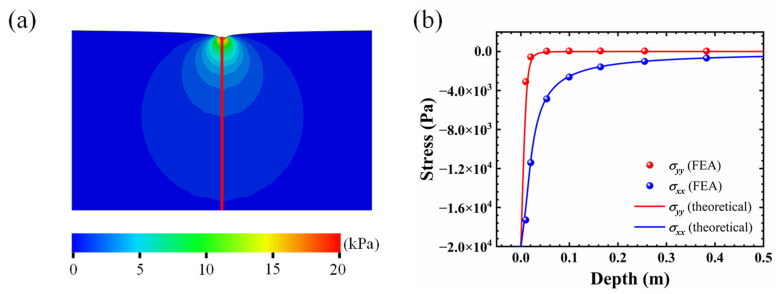
(**a**) The Mises stress distribution from FEA; (**b**) comparisons between theoretical solution and FEA results.

Figure 5a demonstrates the Mises stress distribution obtained from the FEA results. It can be observed that the Mises stress reached the maximum around the compressed region and dramatically decreased to zero when moving away. Figure 5b shows the comparison of *σ_x_* (blue) and *σ_y_* (red) along the red path in Figure 5a. The solid lines and the dots represent the theoretical solutions and FEA results, respectively. The perfect agreement verifies the theoretical solutions. Besides, it shows that the absolute value of *σ_x_* is greater than *σ_y_*. Thus, the minor principal stress *σ_x_* was utilized in the calculation of membrane potential. 

### 3.2. Influence of the Compression Amplitude

**Figure 6:**
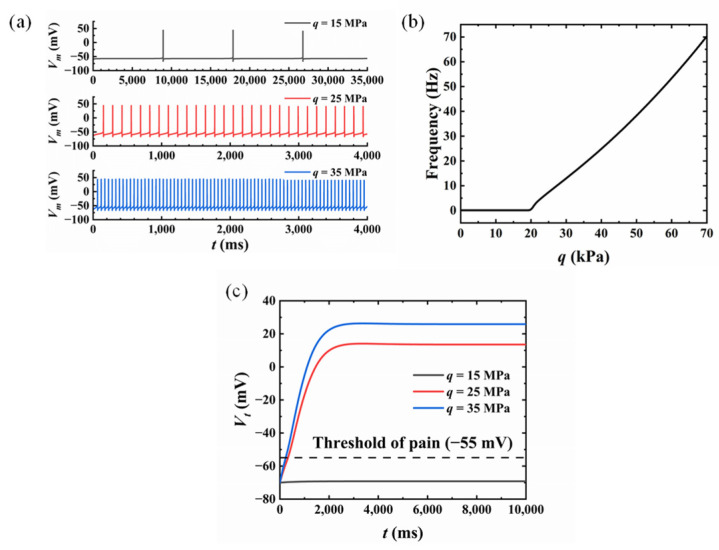
Skin pain sensation with different compression amplitude values: (**a**) membrane potential variation; (**b**) the relationship between compression amplitude and frequency; (**c**) the variation of T-cell output.

Obviously, the level of compression pain is related to the pressure amplitude. Thus, the compression skin pain sensation with different amplitudes is studied in this section, as illustrated in Figure 6. The compression radius was fixed at *a* = 10 mm and the location of the nociceptor was assumed to be at *x* = 1.6 mm, *y* = 0, corresponding with the approximate epidermis thickness of the skin [1]. The nonlinear differential equations of the revised H–H model and the mathematical GCT were solved based on the four-order Runge–Kutta method. Figure 6a demonstrates the membrane potential *V_m_* under different compression amplitude values (*q* = 15 kPa, *q* = 25 kPa, *q* = 35 kPa). It was demonstrated that the membrane potential *V_m_* showed a quasi-periodical variation with time and the stimuli intensity increased with the growing compression amplitude. Figure 6b demonstrates the frequency of *V_m_* was positively related to compression amplitude *q* after surpassing the mechanical threshold *σ*_t_ = 20 kPa. The membrane frequency here was determined by fast-Fourier transformation (FFT). The output of T-cell *V_t_* under different compression values is illustrated in Figure 6c. It was found that *V_t_* increased and reached a plateau finally. The outputs of T-cells caused by *q* = 25 kPa and *q* = 35 kPa both exceeded the threshold value (−55 mV) for pain sensation.

### 3.3. Influence of the Nociceptor Location

**Figure 7:**
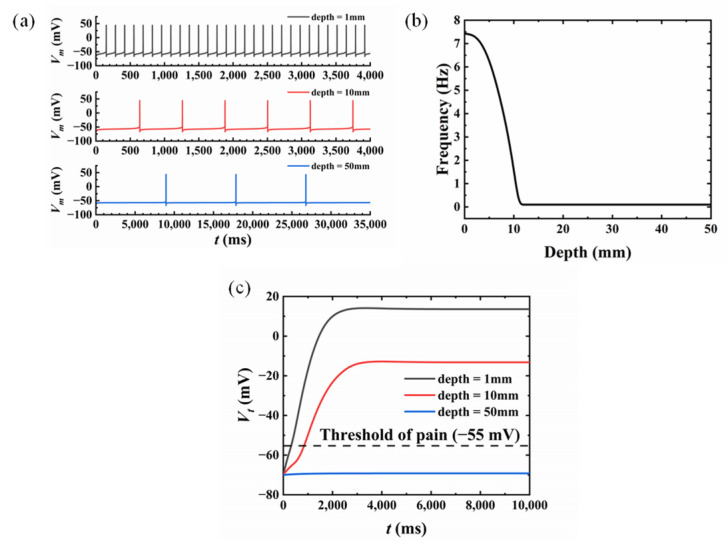
Skin pain sensation with different nociceptor locations: (**a**) membrane potential variation; (**b**) the relationship between frequency and depth of nociceptors; (**c**) the variation of T-cell output.

It can be observed from Figure 5b that the stress on the skin varied with the depth. Thus, it was necessary to study the influence of the location of the nociceptor on the skin pain sensation, as illustrated in Figure 7. The nociceptor was set to locate on the *y*-axis with varying depths to the surface of the skin (1 mm, 10 mm, and 50 mm). The radius and the compression amplitude were taken as *a* = 10 mm and *q* = 25 kPa. It was shown that the frequency of *V_m_* decreased with increasing depth, shown in Figure 7a with the gray line (1 mm), the red line (10 mm), and the blue line (50 mm), respectively. The frequency became stable when the depth continued to increase, corresponding to a stress level less than *σ*_t_ (Figure 7b). The output of T-cell *V_t_* also decreased with the increasing depth of the nociceptor as shown in Figure 7c. With the same compression amplitude of *q* = 25 kPa, the output of T-cell *V_t_* became less than the threshold value when the depth reached 50 mm compared with the other shallower locations (10 mm and 50 mm). In other words, skin with a thinner epidermis is more likely to experience pain sensations. According to our life experience, our hands are indeed more sensitive to pain until the skin grows thick calluses.

## 4. Conclusions

In this paper, a model for compression pain sensation of skin is developed, which includes: (1) a model of skin compression, where the stress distribution on the skin is obtained theoretically based on the Flament solution; (2) a model of transduction, where the electrical signal converted from external mechanical stimuli is analyzed with the revised H–H model; (3) a model of modulation and perception, where the correlation between the nerve impulse and the pain sensation is revealed through the mathematical model of GCT theory. The skin compression model was verified by the perfect agreement between theoretical stress distribution and FEA results. Factors that influence pain sensation were also investigated, including compression amplitude and location of the nociceptor. However, there are some limitations to the model developed in this paper, which still need further investigation. (1) The skin was modeled as a semi-infinite elastic solid in this paper with static loading on the surface, although it has been demonstrated that the viscoelasticity of skin has to be considered in dynamical analysis. (2) The mechanical stimuli compression was uniform in this study, and different kinds of mechanical loadings need to be investigated to account for the complexity of the real situation. (3) The parameters in the model of transduction and the model of modulation and perception have not been compared with experimental results. It is hoped that the parameters could be determined by comparing the mathematical pain sensation model with experimental results.
